# A Cognitively Grounded Measure of Pronunciation Distance

**DOI:** 10.1371/journal.pone.0075734

**Published:** 2014-01-09

**Authors:** Martijn Wieling, John Nerbonne, Jelke Bloem, Charlotte Gooskens, Wilbert Heeringa, R. Harald Baayen

**Affiliations:** 1 Department of Quantitative Linguistics, University of Tübingen, Tübingen, Germany; 2 Center for Language and Cognition Groningen, University of Groningen, Groningen, The Netherlands; 3 Amsterdam Center of Language and Communication, University of Amsterdam, Amsterdam, The Netherlands; 4 Department of Linguistics, University of Alberta, Edmonton, Alberta, Canada; Utrecht University, Netherlands

## Abstract

In this study we develop pronunciation distances based on naive discriminative learning (NDL). Measures of pronunciation distance are used in several subfields of linguistics, including psycholinguistics, dialectology and typology. In contrast to the commonly used Levenshtein algorithm, NDL is grounded in cognitive theory of competitive reinforcement learning and is able to generate asymmetrical pronunciation distances. In a first study, we validated the NDL-based pronunciation distances by comparing them to a large set of native-likeness ratings given by native American English speakers when presented with accented English speech. In a second study, the NDL-based pronunciation distances were validated on the basis of perceptual dialect distances of Norwegian speakers. Results indicated that the NDL-based pronunciation distances matched perceptual distances reasonably well with correlations ranging between 0.7 and 0.8. While the correlations were comparable to those obtained using the Levenshtein distance, the NDL-based approach is more flexible as it is also able to incorporate acoustic information other than sound segments.

## Introduction

Obtaining a suitable distance measure between two pronunciations is important, not only for dialectologists who are interested in finding the relationship between different dialects (e.g., [1]), but also for sociolinguists investigating the effect of political borders on vernacular speech [2], language researchers investigating the typological and genealogical relationships among the world's languages (e.g., [3]), applied linguists attempting to gauge the degree of comprehensibility among related languages [4], and researchers measuring the atypicality of the speech of the bearers of cochlear implants [5]. Furthermore, having a distance measure between word pronunciations enables quantitative analyses in which the integrated effect of geography and sociolinguistic factors can be investigated (e.g., [6]). Standard sociolinguistic analyses focus on whether specific categorical differences are present in the speech of people from different social groups. By using a *measure* of pronunciation difference, we allow more powerful numerical analysis techniques to be used. For these analyses to be meaningful, however, the measurements of pronunciation distance need to match perceptual distances as closely as possible.

There are various computational methods to measure word or pronunciation distance (or similarity), of which the Levenshtein distance has been the most popular [1], [7], [8], [9], [10]. The Levenshtein distance determines the pronunciation distance between two transcribed strings by calculating the number of substitutions, insertions and deletions to transform one string into the other [11]. For example, the Levenshtein distance between two accented pronunciations of the word Wednesday, [wεnzdeI] and [wεn
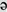
sde] is 3 as illustrated by the alignment in Table 1.

**Table 1:** Basic Levenshtein distance alignment.

w	ε	n		z	d	e	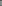
w	ε	n	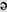	s	d	e	
			1	1			1

A clear drawback of this variant of the Levenshtein distance is that it does not distinguish the substitution of similar sounds (such as [o] and [u]) from more different sounds (such as [o] and [i]). Consequently, effort has been made to integrate more sensitive segment distances in the Levenshtein distance algorithm [1], [12]. As manually determining sensitive segment distances is time-consuming and language-dependent, Wieling and colleagues [13] developed an automatic method to determine sensitive segment distances. Their method calculated the pointwise mutual information between two segments, assigning lower distances between segments which aligned relatively frequently and higher distances between segments which aligned relatively infrequently. Results indicated that the obtained segment distances were acoustically sensible and resulted in improved alignments [14]. Applying the adapted method to the example alignment shown above yields the associated costs shown in Table 2.

**Table 2:** Levenshtein distance alignment with sensitive sound distances.

w	ε	n		z	d	e	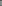
w	ε	n	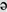	s	d	e	
			0.031	0.020			0.030

While Levenshtein distances correlate well (*r* = 0.67) with perceptual dialect distances between Norwegian dialects [15], there is no cognitive basis to link the Levenshtein distance to perceptual distances (but see [16] for an attempt to adapt the Levenshtein algorithm in line with theories about spoken word recognition). This is also exemplified by the fact that the Levenshtein distance is symmetrical (i.e. the distance between speaker A and B is the same as the other way around), while perceptual dialect distances may also show an asymmetrical pattern [15].

As exposure to language shapes expectations and affects what is judged similar to one's own pronunciation and what is different, we turn to one of the most influential theories about animal and human (discrimination) learning: the model of Rescorla and Wagner [17]. The basic assumption of this model is that a learner predicts an outcome (e.g., the meaning of a word) based on the set of available cues (e.g., the sounds of a word). Depending on the correctness of the prediction, the association strengths between the outcome and the cues are adjusted so that future prediction accuracy improves. Concretely, if an outcome is present together with a certain cue, its association strength increases, while the association strength between an absent outcome and that cue decreases. When an outcome is found together with multiple cues (i.e. when there is cue competition), the adjustments are more conservative (depending on the number of cues). The learning theory of Rescorla and Wagner is formalized in a set of recurrence equations which specify the association strength 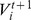 of cue 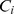 with outcome *O* at time 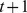 as 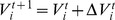, where the change in association strength 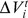 is defined as:
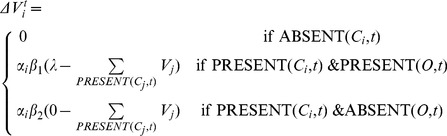
In this definition, 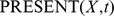 denotes the presence of cue *X* at time *t* and 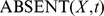 its absence at time *t*. Whenever the cue occurs without the outcome being present, the association strength is decreased, whereas it is increased when both the cue and outcome are present. The adjustment of the association strength depends on the number of cues present together with the outcome. The standard settings for the parameters are 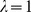, all 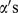 equal, and 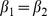.

The Rescorla-Wagner model has been used to explain findings in animal learning and cognitive psychology [18] and more recently, Ramscar and colleagues [19], [20], [21] have successfully used this model in the context of children's language acquisition. For example, Ramscar and colleagues [21] showed that the Rescorla-Wagner model clearly predicted that exposure to regular plurals (such as *rats*) decreases children's tendency to over-regularize irregular plurals (such as *mouses*) at a certain stage in their development.

Danks [22] proposed parameter-free equilibrium equations (i.e. where 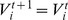) for the recurrence equations presented above: 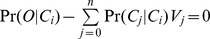, where 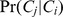 represents the conditional probability of cue 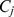 given cue 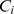, and 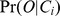 the conditional probability of outcome *O* given cue 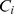. Consequently, it is possible to directly calculate the association strength between cues and outcomes in the stable (i.e. adult) state where further learning does not substantially change the association weights. Baayen and colleagues [23] have proposed an extension to estimate multiple outcomes in parallel. Their ‘naive discriminative learning’ (NDL) approach (implementing the Danks equations [22]) lends itself for efficient computation and is readily available via their R package ‘ndl’. More details about the underlying computations can also be found in [23].

After all association strengths of the adult state are determined, the activation (i.e. activation strength) of an outcome given a set of cues can be calculated by summing the corresponding association strengths. Especially these activations are important for prediction. For example, Baayen and colleagues [23] found that the estimated activation of words correlated well with experimental reaction times to those words.

Here we propose to use naive discriminative learning to determine pronunciation distances. The intuition behind our approach is that a speaker of a certain dialect or language variety is predominantly exposed to speakers who speak similarly, and this input shapes the network of association strengths between cues (in our case, sequences of three sound segments representing the pronunciation, i.e. substrings of the phonetic transcription) and outcomes (in our case, the meaning of the pronounced word) for the speaker. The use of sequences of three segments, so-called trigrams, allows the measure to become sensitive to the adjustments sounds undergo in the context of other sounds, and trigrams have been experimented with in dialectology before [24]. (For comparison, we will also report results when using unigram and bigram cues.) By exposing the speaker to a new pronunciation (in the form of its associated cues) we can measure how well the speaker is likely to understand that pronunciation by inspecting the activation strength of the corresponding outcome. The activation strength of the outcome will depend on the association strengths between the outcome and the cues involved in the pronunciation. If only cues are present which have a high association strength with the outcome, the activation of the outcome will be high, whereas the activation of the outcome will be somewhat lower if one of the cues has a low association strength with the outcome. By calculating the activation strength difference for two different pronunciations of the same word, we obtain a (gradual) measure of pronunciation distance. For example, the word ‘with’ would be highly activated when a native English listener hears [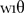]. However, when a Mandarin speaker would incorrectly pronounce ‘with’ as [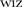], this would result in a somewhat lower activation.

Of course, using an adult state with fixed association weights between cues and outcomes is a clear simplification. Language change is a continuous process and the experience of a listener (i.e. the association weights between cues and outcomes) will obviously be affected by this. However, as the new language experience only makes up a small part of the total language experience of a listener, the effect of the past experience is most important in determining the association weights. As a consequence, and in line with the results of Labov's ([25]: Ch. 4) Cross-Dialectal Comprehension (CDC) studies (which evaluated how well American English speakers understand speakers from their own and other regions), our model will yield lower meaning activations (i.e. more misunderstandings) when sound change is in progress (i.e. the original sound segments will have a higher association strength with the meaning than the new sound segments). In similar fashion, our model predicts higher meaning activations for pronunciations closer to one's own pronunciation variant (i.e. the “local advantage”). We also emphasize that our model is able to capture differences in understandability per word (as each word has its own frequency of occurrence) – which might explain Labov's finding that certain sounds are not always correctly identified, even if they are characteristic of local speakers ([25]: pp. 84–85).

Furthermore, the model we propose is general, as it does not focus on a selection of linguistic features (such as vowels), but takes into account all sound (sequences) in determining the understandability of a certain pronunciation.

Besides being grounded in cognitive theory of competitive reinforcement learning, a clear benefit of this approach is that the pronunciation distances obtained do not need to be symmetrical, as they depend on the association strengths between cues and outcomes, which are different for every speaker. This is illustrated in Section 2.2 below.

To evaluate the effectiveness of this approach, we conducted two experiments. The first experiment focused on investigating foreignness ratings given by native American English (AE) speakers when judging accented English speech, while the second experiment focused on the asymmetric perceptual distances of Norwegian dialect speakers.

As we noted in the introduction, the Levenshtein distance has been applied to pronunciation transcriptions to assay the degree to which non-local pronunciations sound “different” from local ones (in dialectology, see [1]), but also to predict the comprehensibility of other language varieties (in applied sociolinguistics, see [4]). Since pronunciations may sound non-native or non-local without suffering in comprehensibility, one might suspect that the two notions are not the same, even if they are clearly related. In the present paper we construct a model of an artificial listener to discriminate well enough between words given sound trigrams, which is essentially a comprehension task. But we shall evaluate the same model on how well it predicts human judgments of how similar the speech is to one's own pronunciation (i.e. how native-like foreign accents sound, or how close a pronunciation is to one's own dialect). To the degree to which these experiments succeed, we may conclude that the degree of comprehensibility is largely the same as the degree of nativeness (or localness).

## Materials and Methods

### 1. Accented English speech

#### 1.1. Material: the Speech Accent Archive

The Speech Accent archive [26] is digitally available at http://accent.gmu.edu and contains a large sample of speech samples in English from people with various language backgrounds. Each speaker read the same paragraph of 69 words (55 of which are unique) in English:

*Please call Stella. Ask her to bring these things with her from the store: six spoons of fresh snow peas, five thick slabs of blue cheese, and maybe a snack for her brother Bob. We also need a small plastic snake and a big toy frog for the kids. She can scoop these things into three red bags, and we will go meet her Wednesday at the train station.*

All speech samples were transcribed by three phonetically trained transcribers (consensus was reached in the few cases where the transcriptions differed; [26]) according to the International Phonetic Alphabet (IPA). The transcriptions include diacritics, and the associated audio files are available. For this study, we extracted 395 transcribed speech samples and their audio from the Speech Accent Archive. The total number of native U.S.-born English speakers in this dataset was 115. The remaining 280 speech samples belonged to speakers with a different native language or who were born outside of the United States.

#### 1.2. Obtaining NDL-based pronunciation distances

For every transcribed pronunciation, we extracted all possible sets of sequences of three sound segments (diacritics were ignored, and a separate segment was added to mark word boundaries) as cues. To model a native AE listener, we randomly selected about half (i.e. 58) of the native AE speakers. We used their pronunciations to generate the pronunciation cues, and paired these with meanings as outcomes (i.e. the pronunciation trigrams were linked to the corresponding meanings). We used only half of the native speakers for the listener model in order to prevent overfitting, i.e. learning the peculiarities of the speakers rather than the features of native American English. The pronunciation of the other half of the speakers is used to represent average American English speech to which the pronunciation of individual speakers is compared. (While we could have used the speech of a single speaker for the listener model and the speech of another individual speaker to represent native American English speech, this would have biased the model to the specific dialectal variants of these speakers.) As the association strength between cues and outcomes depends on the frequency with which they co-occur, we extracted word frequency information from the Google N-Gram Corpus [27]. The total frequency of each meaning outcome was equally divided among all different pronunciations associated with it. For example, if the frequency of the word ‘frog’ equals 580,000, the frequency of each of the 58 pronunciations was set to 10,000. We then estimated the weights of the model using the ‘ndl’ package in R (version 0.2.10) which implements the Danks equations [23] introduced above. The resulting network of association strengths between pronunciation cues and meaning outcomes represents a native AE listener. As an example, Table 3 shows part of the input used for estimating the weights and Table 4 shows the association strengths obtained after the weights have been estimated (i.e. the ‘adult’ association weights of a native AE listener).

**Table 3:** Part of the table used for estimating the association strengths. The ‘#’ marks the word boundary.

Speaker	Outcome	Pronunciation	Cues	Frequency
english23	with	[w 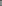 θ]	#w 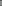 , w 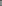 θ, 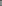 θ#	28,169,384
english167	with	[w 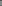 ð]	#w 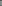 , w 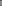 ð, 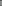 ð#	28,169,384
english23	her	[h 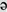 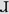 ]	#h 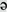 , h 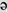 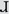 , 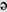 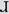 #	852,131
english167	her	[ 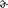 ]	# 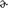 #	852,131

**Table 4:** The association strengths for the cues and outcomes in Table 1 for our simulated native AE listener after these have been estimated on the basis of the input of 58 randomly selected native AE speakers.

Cue	Association strength for ‘with’	Association strength for ‘her’
#w 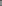	0.2519	0.0000
w 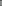 θ	0.3738	0.0000
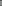 θ#	0.3738	0.0000
w 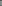 ð	0.3741	0.0000
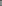 ð#	0.3741	0.0000
#h 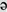	0.0000	0.4973
h 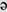 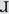	0.0000	0.2433
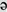 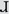 #	0.0000	0.2594
# 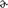 #	0.0000	1.0000

It is clear from Table 4 that the cues found together with a certain outcome generally have a positive value. The more likely it is the cue is found together with the associated outcome (and, crucially, not with other outcomes), the higher the association strength between the two will be.

Given the table of association strengths representing a simulated native AE listener, it is straightforward to determine the activations of each outcome for a certain pronunciation (converted to cues) by summing the association strengths between the cues in the pronunciation and the outcome. The top half of Table 5 shows that the pronunciations of native AE speakers strongly activate the corresponding outcome (the values are equal or very close to the maximum of 1).

**Table 5:** The activations of different outcomes on the basis of the association strengths between the cues and outcomes for our simulated native AE listener (shown in Table 2).

Speaker	Outcome	Pronunciation	Cues	Activation of outcome
english23	with	[w 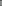 θ]	#w 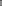 , w 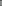 θ, 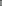 θ#	0.9995
english167	with	[w 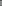 ð]	#w 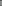 , w 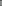 ð, 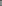 ð#	1.0000
english23	her	[h 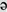 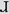 ]	#h 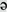 , h 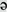 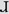 , 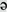 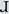 #	1.0000
english167	her	[ 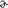 ]	# 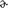 #	1.0000
mandarin10	with	[w 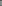 z]	#w 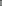 , w 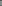 z, 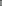 z#	0.2519
serbian10	her	[x 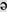 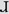 ]	#x 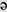 , x 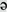 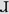 , 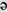 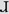 #	0.2594

Of course, we can also use the association strengths (of the simulated native AE listener) to calculate the activations for accented speech. The bottom part of Table 5 clearly shows that accented speech results in lower activations (and thus reduced understanding), compared to the pronunciations of native AE speakers (shown in the top part of Table 5). In some cases, a foreign speaker might use a cue which would never be used by a native AE speaker (such as ‘#x
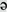
’ in Table 5). As these cues were not encountered during the estimation of the model, no association strengths have been set for those cues and, consequently, their values do not contribute to the activation of the outcome.

To determine pronunciation distances with respect to native American English, we exposed our model of a native AE speaker to both native American English speech as well as accented English speech and investigated the activation differences of the meaning outcomes. We used the following procedure:

1. For each of the native American English speakers not considered when constructing the listener model (i.e. the remaining 57 native AE speakers), we calculated the activation of the listener model for each of the 55 different meaning outcomes (i.e. all unique words in our dataset). Whenever an outcome occurred more than once (such as ‘we’, which occurs twice in the paragraph of text), we averaged the activations associated with the corresponding pronunciations (i.e. the associated cues). For each outcome, we subsequently averaged the activations across all 57 speakers. This is our baseline and can be interpreted as the activations (for 55 individual meanings) of our native AE listener model when being exposed to the speech of an *average* native AE speaker.
2. For each individual speaker (mostly non-native, see below), we obtained the activations of our native AE listener model for each of the 55 meanings. Again, whenever an outcome occurred more than once, we averaged the activations associated with the corresponding pronunciations.
3. For each individual speaker, we calculated the activation difference compared to the baseline for all 55 meanings separately. We then averaged these activation differences across the 55 meanings. This resulted in a single value for each speaker and represents the NDL-based pronunciation distance with respect to an average native AE speaker.

As the specific sample of speakers used for estimating the native American English listener model may influence the results, we repeated the random sampling procedure (in which 58 speakers were selected whose pronunciations were used to estimate the listener model) 100 times to generate 100 slightly different native AE listener models. Obviously, this also resulted in a change of the remaining 57 speakers who were used to represent an average AE speaker (see step 1, above). Consequently, we obtained 100 (slightly different) NDL-based pronunciation distances for each individual speaker compared to an average AE speaker.

#### 1.3. Validating automatically obtained foreignness ratings

We evaluated the computed pronunciation distances by comparing them to human native-likeness ratings. For this purpose, we developed an online questionnaire for native U.S. English speakers. In the questionnaire, participants were presented with a randomly ordered subset of 50 speech samples from the Speech Accent Archive. We did not include all speech samples, as our goal was to obtain multiple native-likeness-judgments per sample. For each speech sample, participants had to indicate how native-like each speech sample was. This question was answered using a 7-point Likert scale (ranging from 1: very foreign sounding to 7: native AE speaker). Participants were not required to rate all samples, but could rate any number of samples.

Of course, more advanced methods are possible to measure native-likeness, such as indirect measures which assess the understandability of the accented pronunciations in a certain context (cf. [25: Ch. 4]). However, as our dataset was limited to a small fixed paragraph of text, we used a simple rating approach which, nevertheless, resulted in consistent ratings (see results, below).

Via e-mail and social media we asked colleagues and friends to forward the online questionnaire to people they knew to be native AE speakers. In addition, the online questionnaire was advertised on Language Log by Mark Liberman. Especially that announcement led to an enormous amount of responses. As a consequence, we replaced the initial set of 50 speech samples five times with a new set to increase the number of speech samples for which we could obtain native-likeness ratings. As there was some overlap in the native AE speech samples present in each set (used to calibrate the ratings), the total number of unique samples presented for rating was 286, of which 280 were samples from speakers who were not born in the U.S.

### 2. Norwegian dialects

#### 2.1. Material

The Norwegian dialect material is taken from the study of Gooskens and Heeringa [15], who perceptually evaluated the Levenshtein distance on the basis of IPA transcribed audio recordings of 15 Norwegian dialect speakers reading the fable “The North Wind and the Sun” (containing 58 unique words). The original dataset was created by Jørn Almberg and Kristian Skarbø and is available at http://www.ling.hf.ntnu.no/nos. The transcriptions (including diacritics) were made by the same person, ensuring consistency. Perceptual distances (reported in Table 1 of [15]) were obtained by asking 15 groups of high school pupils (in the corresponding dialect areas) to rate all 15 dialectal audio samples on a scale from 1 (similar to own dialect) to 10 (not similar to own dialect). Perceptual dialect distances were then calculated by averaging these ratings per group.

#### 2.2. Methods

Following the same procedure as described in Section 1.2, we converted the pronunciations for each of the 15 speakers in our sample to cues consisting of three sequential sound segments (diacritics were ignored, and a separate segment was added to mark word boundaries). The word frequencies were extracted from a Norwegian word frequency list (on the basis of subtitles and obtained from http://invokeit.wordpress.com/frequency-word-lists).

To determine pronunciation distance between dialects *D_i_* and *D_j_* from the perspective of a listener of dialect *D_i_*, we used the following procedure:

1. We estimated the NDL model (i.e. resulting in a specific weight matrix associating cues with outcomes) using the cues on the basis of the pronunciations from the speaker of dialect *D_i_*. This model can be seen as representing an experienced listener (*L_i_*) of dialect *D_i_*.
2. We expose *L_i_* to the cues on the basis of the pronunciations from dialect *D_i_* and measure the activation of each of the corresponding 58 meaning outcomes. (Because we only had a single speaker in our sample for each dialect, we could not use separate pronunciations for estimating the listener model and representing the speaker.). Whenever an outcome occurred more than once (some words were repeated), we averaged the activations associated with the corresponding pronunciations (i.e. the associated cues). These activations are used as the baseline, and can be interpreted as the activations (for the 58 individual meanings) of *L_i_* when being exposed to speech of its own dialect.
3. We expose *L_i_* to the cues on the basis of the pronunciations of another dialect *D_j_* and measure the (averaged, when a word occurred more than once) activation of each of the corresponding 58 meaning outcomes.
4. For all 58 individual meaning outcomes, we calculated the difference between the activations of *L_i_* for *D_j_* and the baseline *D_i_* and average these 58 differences to get a single value representing the NDL-based pronunciation distance between *D_i_* and *D_j_* (from the perspective of *L_i_*).

The above procedure is repeated for all combinations of *D_i_* and *D_j_* resulting in 210 NDL-based pronunciation distances (15×15, but the 15 diagonal values are excluded as they are always equal to 0). Table 6 shows these distances for a set of three Norwegian dialects. Note that the NDL-based pronunciation distances between these dialects are clearly asymmetric. The dialect of Bjugn is closer to the dialect of Bergen from the perspective of Bergen (0.545) than the dialect of Bergen is from the perspective of Bjugn (0.559).

**Table 6:** Part of the NDL-based Norwegian dialect pronunciation distances.

	Bergen	Bjugn	Bodø
Bergen	X	0.545	0.584
Bjugn	0.559	X	0.319
Bodø	0.574	0.314	X

To evaluate these distances, we correlated them with the corresponding perceptual distances (obtained from [15]).

## Results

### 1. Results for accented English speech

A total of 1143 native American English participants filled in the questionnaire (658 men: 57.6%, and 485 women: 42.4%). Participants were born all over the United States, with the exception of the state of Nevada. Most people came from California (151: 13.2%), New York (115: 10.1%), Massachusetts (68: 5.9%), Ohio (66: 5.8%), Illinois (64: 5.6%), Texas (55: 4.8%), and Pennsylvania (54: 4.7%). The average age of the participants was 36.2 years (SD: 13.9) and every participant rated on average 41 samples (SD: 14.0). Every sample was rated by at least 50 participants and the judgments were consistent (Cronbach's alpha: 0.853).

**Figure 1:**
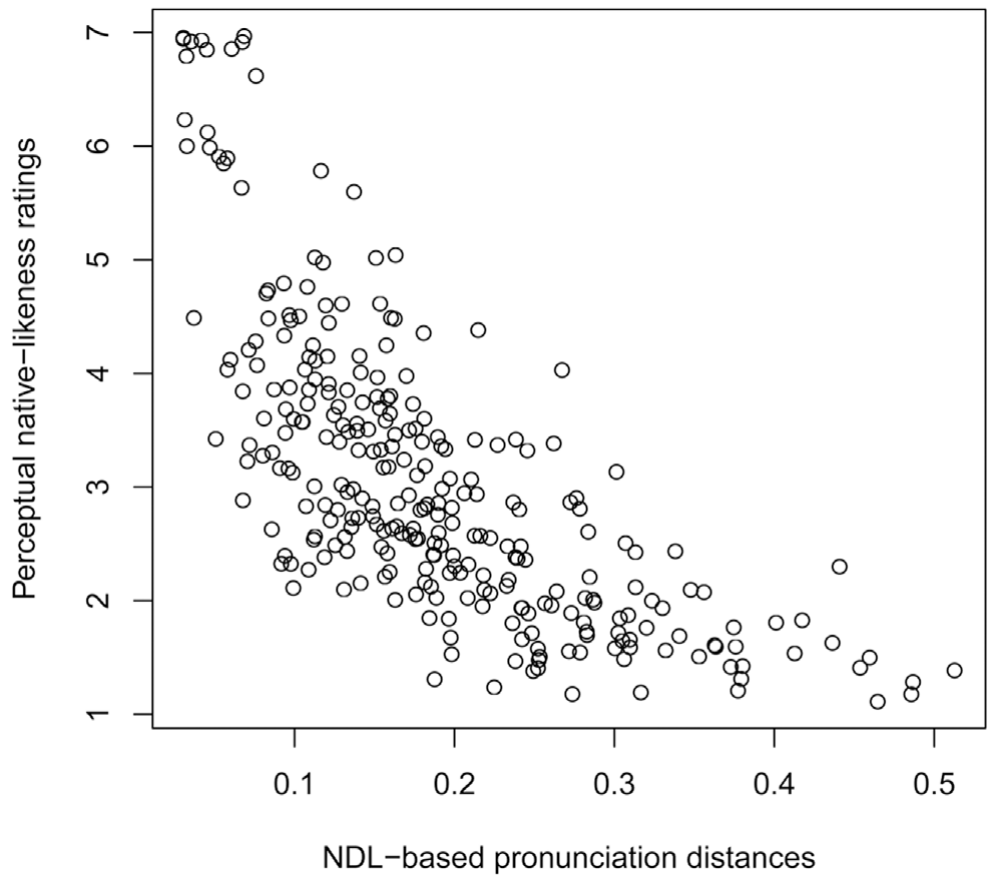
Logarithmic relationship between NDL-based pronunciation distances and perceptual distances.

To determine how well our NDL-based pronunciation distances on the basis of trigram cues matched the native-likeness ratings, we calculated the Pearson correlation *r* between the averaged ratings and the NDL-based pronunciation distances for the 286 speakers. Since we had 100 sets of NDL-based pronunciation distances (based on 100 different random samplings of the native American English speakers used to estimate the model), we averaged the corresponding correlation coefficients, yielding an average correlation of *r* = −0.72 (*p*<0.001). Note that the direction of the correlations is negative as the participants indicated how *native-like* each sample was, while the NDL-based pronunciation distance indicates how foreign a sample is. As a scatter plot clearly revealed a logarithmic relationship (see Figure 1), we log-transformed the NDL-based pronunciation distances, increasing the correlation to *r* = −0.80 (*p*<0.001). The logarithmic relationship suggests that people are relatively sensitive to small differences in pronunciation in judging native-likeness, but as soon as the differences have reached a certain magnitude (i.e. in our case an NDL-based pronunciation distance of about 0.2) they hardly distinguish them anymore. The sensitivity to small differences is also illustrated by the (slight) increase in performance when trigram cues are used which incorporate diacritics. In that case, the correlation strength increases to *r* = −0.75 (*r* = −0.82 for the log-transformed NDL-based pronunciation distances). These results are comparable with the performance of the Levenshtein distance when applied to this dataset (*r* = −0.81, *p*<0.001 for the log-transformed Levenshtein distance; unpublished data). In fact, the Levenshtein distances and the NDL-based pronunciation distances also correlate highly, *r* = 0.89 (*p*<0.001).

We should note that this correlation is close to how well individual raters agree with the average native-likeness ratings (on average: *r* = .84, *p*<.0001). Consequently, the NDL-based method is almost as good as a human rater, despite ignoring suprasegmental pronunciation differences (such as intonation).

Figure 1 also shows that pronunciations which are perceived as native (i.e. having a rating very close to 7), may correspond to NDL-based pronunciation distances greater than 0. In this case, the NDL-based method classifies certain native-like features as being non-native. This may be caused by our relatively small sample of only 58 speakers whose pronunciations were used to model the native AE listener. Real listeners have much more experience with their native language, and therefore can more reliably distinguish native-like from foreign cues.

The aforementioned results are all based on using trigram cues. When using unigram cues instead, the correlation between the perceptual native-likeness ratings and the NDL-based pronunciation distances dropped to *r* = −0.54 (log-transformed: *r* = −0.57). When using bigram cues, the performance was almost on par with using trigram cues (*r* = −0.69, log-transformed: *r* = −0.79). Using unigram and/or bigram cues together with trigram cues did not affect performance, as these simpler cues are not discriminative in the presence of trigram cues.

### 2. Results for Norwegian dialects

The correlation between the NDL-based pronunciation distances and the perceptual distances was *r* = 0.68 (*p*<0.001), which is comparable to the correlation Gooskens and Heeringa [15] reported on the basis of the Levenshtein distance (i.e. *r* = 0.67). Similar to the first study, log-transforming the NDL-based pronunciation distances increased the correlation strength to *r* = 0.72 (*p*<0.001). In line with the results for the accent data, the Levenshtein distances and the NDL-based pronunciation distances correlate highly, *r* = 0.89 (*p*<0.001).

The aforementioned results are all based on using trigram cues. Using unigram cues instead of trigram cues severely reduced performance (*r* = 0.10, log-transformed: *r* = 0.31), whereas using bigram cues was almost as good as using trigram cues (*r* = 0.67, log-transformed: *r* = 0.71). Similar as before, adding unigram and/or bigram cues to the trigram cues did not really improve performance. In contrast to the accent data, incorporating diacritics in the cues also did not help; the correlation then dropped to *r* = 0.65 (log-transformed: *r* = 0.66). This is likely caused by the relatively small dataset.

## Discussion

In the present paper we have shown that pronunciation distances derived from naive discriminative learning match perceptual accent and dialect distances quite well. While the results were on par with those on the basis of the Levenshtein distance, the advantage of the present approach is that it is grounded in cognitive theory of comprehension based on fundamental principles of human discrimination learning. Furthermore, the Levenshtein distance is theoretically less suitable for modeling the degrees of difference in the perception of non-local and non-native speech because it is a true distance, i.e. always symmetric, while perceptions of similarity may also be asymmetric [15]. The NDL-based approach naturally generates asymmetrical distances.

We noted above that the task of recognizing words based on phonetic cues is essentially a comprehensibility task. A second contribution of the present paper is therefore to demonstrate that models constructed to comprehend local speech automatically assign scores of non-nativeness (or of non-localness among dialects) in a way that models native speakers judgments.

One may wonder why the NDL-based method only slightly improved upon the results of the Levenshtein distance for the Norwegian dataset, especially since that dataset is characterized by asymmetric perceptual distances. We note here that the 15 NDL models (one for each listener) are only based on the pronunciation of a single speaker. Consequently, it does not take into account the variation within each dialect (taken into account by listeners living in the dialect area), which would have allowed for more precise estimates of the association weights. A general limitation is that Gooskens and Heeringa [15] already indicated that intonation is one of the most important characteristics in Norwegian dialects, and no such cues have been used here (as these were not available to us), thereby limiting the ability to detect relevant asymmetries. Nerbonne and Heeringa ([28]: 563–564), on the other hand, speculate that there is a limit to the accuracy of validating pronunciation difference measures on the basis of aggregate judgments of varietal distance. If one supposes that poorer measures are noisier – but not more biased – than better ones, then the noise will simply be eliminated in examining large aggregates. If this is right, we cannot expect to change mean differences by adopting more accurate measurements. They suggest that improved validation will therefore have to focus on smaller units such as individual words.

While we have not explored this in the present paper, another important advantage of the NDL approach is that cues are not only restricted to phonetic segments. Cues with respect to pronunciation speed or other acoustic characteristics (such as intonation) can be readily integrated in an NDL model (e.g., linking cues representing different intonation patterns to the individual meanings). A problem of the NDL method, however, is that it only accepts discrete cues. A continuous measurement therefore needs to be discretized to separate cues, and this introduces a subjective element in an otherwise parameter-free procedure.

As our datasets only consisted of a few dozen words, our model was highly simplified compared to the cognitive model of a human listener who will have access to thousands of words. It is nevertheless promising that pronunciation distances on the basis of our simplified models match perceptual distances at least as well as current gold standards.
